# DSE-YOLO11: Dynamic feature adaptation for key traffic element detection in complex road scenes

**DOI:** 10.1371/journal.pone.0350328

**Published:** 2026-06-01

**Authors:** Yange Chen, Baohua Guo, David Bassir, Anthony Sigama, Yuandi Zhang

**Affiliations:** 1 School of Energy Science and Engineering, Henan Polytechnic University, Jiaozuo, Henan, China; 2 Jiaozuo Engineering Research Center of Road Traffic and Transportation, Henan Polytechnic University, Jiaozuo, Henan, China; 3 Smart Structural Health Monitoring and Control Laboratory, DGUT-CNAM, Dongguan University of Technology, Dongguan, China; 4 ENS -Paris-Saclay University, Centre Borelli, UMR CNRS 9010, Gif-sur-Yvette, France; 5 Faculty of Science, Engineering and Agriculture, University of Venda, Thohoyandou, Limpopo, South Africa; 6 International Business College, Dongbei University of Finance and Economics, Dalian, Liaoning, China; Nanjing Forestry University, CHINA

## Abstract

Accurate detection of key traffic elements in complex road scenes is critical for autonomous driving and intelligent transportation systems. However, existing lightweight detectors often suffer from missed detections under small targets, large-scale variations, and cluttered backgrounds. To address these challenges, we propose DSE-YOLO11, a RAD-oriented lightweight adaptation of YOLO11n that integrates DynamicConv, SlimNeck, and EMA in a stage-wise collaborative manner. The main contribution lies not in introducing entirely new primitive modules, but in developing a task-specific integration strategy for improving detection robustness in complex road scenes. Specifically, a dynamic convolution-based backbone improves local feature modeling and representation of irregular and small-scale targets. A lightweight neck strengthens cross-scale feature interaction while reducing redundant fusion overhead. Additionally, an efficient attention mechanism suppresses background interference and enhances responses to key regions. Experiments on the RAD dataset show that DSE-YOLO11 improves recall from 0.744 to 0.811 and mAP50 from 0.810 to 0.856, while maintaining 2.96M parameters and 7.1 GFLOPs. These gains are practically meaningful because they indicate fewer missed detections of small, low-contrast, and safety-relevant traffic elements in complex road scenes. Additional experiments on BDD100K provide preliminary external support, although broader validation is still needed.

## 1. Introduction

Intelligent transportation systems and autonomous driving technologies rely heavily on accurate and timely perception of complex road environments. Reliable detection of key traffic elements is essential not only for driving assistance and downstream decision-making, but also for broader transportation safety analysis and risk-oriented traffic management. Recent transportation studies have shown that complex road environments often involve heterogeneous participants, uncertain interactions, and safety-critical behaviors, which increase the demand for accurate scene understanding [1–4]. At the same time, resilient transportation operation also depends on timely perception of road conditions and surrounding traffic elements [5]. In this study, six representative categories of key traffic elements are considered, including light multi-purpose vehicles, heavy vehicles, road damage, unsurfaced roads, pedestrians, and speed bumps. These elements typically exhibit multi-category coexistence, considerable scale variation, and large intra-class appearance differences. In particular, under forward-looking monocular vision, small-scale and low-contrast targets are highly prone to missed detections. For road-safety-oriented perception tasks, missed detections can be especially problematic because undetected hazards may directly affect downstream perception and decision-making. Therefore, improving recall while maintaining low model complexity and efficient inference remains an important objective in road-scene traffic-element detection.

Deep learning-based object detection methods have become the mainstream. These studies indicate that lightweight backbone design and deployment-aware optimization can improve practical applicability for road-scene perception. Two-stage detectors, such as R-CNN [6] and Faster R-CNN [7], generally provide strong detection accuracy but often require relatively complex inference pipelines and higher computational cost. In contrast, one-stage detectors, represented by SSD [8] and YOLO series [9], are more suitable for real-time deployment because of their end-to-end design and high inference efficiency. In recent years, lightweight YOLO-based models have achieved notable progress through improvements in backbone design [10], feature fusion strategies [11], and training optimization [12]. Despite these advances, lightweight detectors still face substantial challenges in complex road scenes, especially when handling small objects, drastic scale variations, and weak target-background contrast. Model compression and lightweight design can reduce computational cost, but they may also weaken feature representation ability and lead to degraded recall for difficult traffic elements [13].

To improve the robustness of lightweight detectors, existing studies have explored several complementary directions. One line of work focuses on lightweight object detection in road scenes through backbone simplification, pyramid optimization, attention enhancement, and deployment-oriented design. For example, L-YOLO [14] improves the trade-off between accuracy and efficiency by introducing a lightweight backbone, a small-object detection layer, loss-function optimization, and pruning. ESPPNet [15] enhances real-time traffic detection through progressive spatial pyramid pooling and multi-scale feature enhancement. Lightweight YOLOv8-based traffic sign detectors [16] have also emphasized edge deployment, while lightweight YOLOX-based networks [17] combine simplified feature pyramid design with attention compensation to improve speed-accuracy balance. These studies indicate that lightweight backbone design and efficiency-oriented optimization can improve practical applicability, but many of them focus on limited traffic categories or generic lightweight optimization rather than robust recall-oriented detection of diverse key traffic elements in complex road scenes.

Another important direction is multiscale feature fusion and feature enhancement for small objects and targets with large-scale variation. Prior studies have shown that feature pyramid optimization, additional detection branches, and scale-aware feature enhancement can improve the representation of difficult targets. For instance, Yuan et al. [18] proposed the Multi-Feature Pyramid Network (MFPNet) to improve adaptability to inter-object scale variations. Qi et al. [13] developed SODNet to strengthen feature extraction for small-scale traffic objects, and Xu et al. [19] missed detections of small objects by incorporating an attention mechanism and a novel convolutional module. YOLO-BS [20] introduced an additional small-object detection layer to enrich high-resolution feature representation and improve the detection of small traffic signs under scale variation. Related efforts in remote sensing, such as MEFE-Net [21], have also demonstrated the value of multiscale edge-aware extraction and feature calibration for improving scale-specific representation and localization. Although these approaches enhance feature representation for small and scale-variant targets, they often do not fully address the combined challenges of background clutter, weak target-background contrast, and lightweight deployment in complex road scenes.

In addition to multiscale fusion, adaptive and attention-based feature enhancement has increasingly been adopted to improve discriminative representation under challenging imaging conditions. In remote-sensing detection, adaptive multiscale fusion has been used to enhance feature interaction across resolutions and improve scale-aware representation [22]. MANet [23], for example, combines multiscale context extraction with adaptive fusion for semantic segmentation in remote-sensing images. In UAV-oriented detection, AAPW-YOLO [24] integrates adaptive convolution with reconstructed feature fusion to strengthen feature extraction for small objects under scale variation and background interference. Similarly, KL-YOLO [25] emphasizes lightweight adaptive global feature enhancement for small-object detection in low-altitude remote-sensing imagery, and ASF-YOLO [26] uses attentional scale-sequence fusion to improve the selective aggregation of scale-sensitive features. These studies suggest that adaptive modeling and attention can improve feature selectivity under scale variation and background interference. However, many of these methods were developed for remote-sensing, UAV, or segmentation tasks, and their direct applicability to lightweight, recall-oriented detection of multi-category key traffic elements in complex road scenes remains limited. In addition, some methods rely on multiple enhancement modules, which may increase structural complexity and reduce deployment efficiency on resource-constrained platforms.

Taken together, existing lightweight detectors still face three major limitations in practical road-scene traffic-element detection: insufficient local feature modeling for small or irregular targets, inefficient cross-scale interaction under large scale variation, and inadequate emphasis on critical regions in cluttered backgrounds. Rather than proposing new primitive operators, this work presents a RAD-oriented lightweight redesign of YOLO11n in which established modules are integrated according to their complementary roles in the detection pipeline.

To address these issues, we developed DSE-YOLO11, a lightweight adaptation framework for key traffic element detection in complex road scenes. Built upon the YOLO11n baseline, the framework is designed to improve detection robustness under scale variation, weak target-background contrast, and background clutter. It enhances lightweight detection through adaptive local feature modeling, more effective cross-scale information interaction, and improved emphasis on critical target regions. It is evaluated primarily on the RAD dataset, with additional external analysis on BDD100K. The main contributions of this study are as follows:

1. We develop a RAD-oriented lightweight adaptation of YOLO11n to address three coupled challenges in complex road scenes: weak local representation of small or irregular anomalies, inefficient cross-scale interaction under large-scale variation, and strong background interference.
2. We propose a stage-wise integration strategy in which DynamicConv, SlimNeck, and EMA are deployed in different parts of the detector according to their functional complementarity, rather than being introduced as isolated generic add-ons.
3. Extensive experiments and ablation studies demonstrate that this application-driven collaborative redesign achieves a better recall-accuracy-complexity trade-off on the RAD dataset than the baseline YOLO11n and several representative detectors.
4. We evaluate the proposed method on the RAD benchmark and additionally examine its external adaptability on BDD100K. The results show that DSE-YOLO11 improves recall and mAP50 relative to YOLO11n on RAD and achieves stronger transfer-learning performance than the baseline on BDD100K.

## 2. Materials and methods

### 2.1. DSE-YOLO11

DSE-YOLO11 does not introduce new atomic operators. Its methodological contribution lies in a RAD-oriented reorganization of established modules within YOLO11n, where DynamicConv, SlimNeck, and EMA are assigned to the backbone, neck, and detection features according to their functional roles.

YOLO11 includes variants of different capacities (n/s/m/l/x). In this study, YOLO11n was selected as the baseline because it offers a favorable lightweight starting point for architecture adaptation. YOLO11 is mainly composed of the backbone network, neck network, and detection head. Compared with its predecessor models, YOLO11 replaces the original C2f module in its backbone network with the C3k2 module. Specifically, this module optimizes gradient propagation and multi-scale feature fusion via smaller convolutional kernels, thereby effectively reducing overall computational complexity while preserving robust feature extraction. Furthermore, the C2PSA module incorporates a spatial attention mechanism that guides the model to focus more precisely on critical target regions within the image, markedly boosting its detection of small-scale objects and occluded instances. The Anchor-Free detection head simplifies training and significantly improves the model’s generalization across different scenarios. In summary, YOLO11 integrates the aforementioned modular innovations to achieve synergistic improvements in detection accuracy, inference speed, and emerges as a state-of-the-art efficient solution for next-generation real-time object detection tasks.

**Fig 1:**
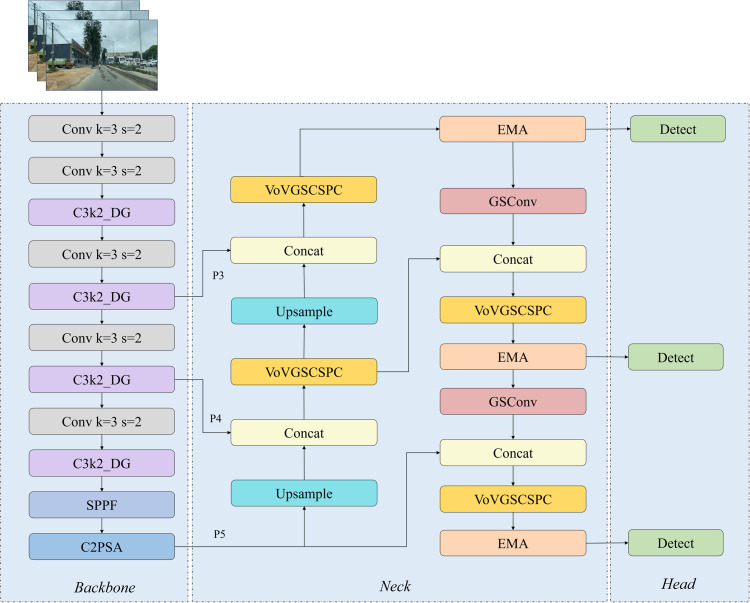
The network architecture of DSE-YOLO11.

This section presents the DSE-YOLO11 architecture, which is developed based on the canonical YOLO11 network framework and incorporates three key optimization strategies: enhancing the C3k2 module, improving the efficiency of multi-scale feature fusion, and integrating a targeted attention mechanism. The core objective of this architecture is to address the critical challenges inherent in road scene detection for autonomous driving, including significant variations in target scale, low detection accuracy, and missed target detections. The DSE-YOLO11 architecture is shown in Fig 1.

As illustrated in Fig 1, the C3k2 module within the backbone network is replaced with the modified C3k2_DG module. This module substitution enhances the model’s adaptability to input features with diverse scales, thereby improving overall detection accuracy for complex traffic elements. In the backbone, the original C3k2 blocks of YOLO11n were replaced with the proposed C3k2_DG blocks at four feature extraction stages. Specifically, C3k2_DG was inserted after the P2/4, P3/8, P4/16, and P5/32 downsampling layers. The nominal output channels of these four stages were set to 256, 512, 512, and 1024, respectively, before width scaling. In the neck, SlimNeck is implemented using VoV-GSCSPC and GSConv. After the first upsampling and concatenation with the backbone P4 feature, a VoV-GSCSPC block with 512 output channels is used. After the second upsampling and concatenation with the backbone P3 feature, a VoV-GSCSPC block with 256 output channels is used to generate the P3/8 feature map. In the subsequent bottom-up path, two GSConv layers are used for downsampling, with kernel size = 3 and stride = 2, and output channels of 256 and 512, respectively. These are followed by VoV-GSCSPC blocks with 512 and 1024 output channels to generate the P4/16 and P5/32 feature maps. This integration effectively preserves implicit inter-channel correlations while limiting time complexity. It also refines multi-scale feature fusion and improves mAP. Additionally, the Efficient Multi-scale Attention (EMA) module is inserted immediately after the three neck outputs used for detection, i.e., the P3/8, P4/16, and P5/32 feature maps. The corresponding channel dimensions are 256, 512, and 1024, respectively, and the EMA-refined features are then directly fed into the final detection head. By reorganizing the channel and batch dimensions, this mechanism enhances the model’s ability to extract and integrate critical features, thereby effectively improving recall performance. The parameter configuration and embedding location of the improved module are detailed in Table 1.

**Table 1:** Concise configuration of DSE-YOLO11.

Component	Position	Output channels	Key setting
C3k2_DG	Backbone stage 1	256	DynamicConv experts = 4
C3k2_DG	Backbone stage 2	512	
C3k2_DG	Backbone stage 3	512	
C3k2_DG	Backbone stage 4	1024	
VoV-GSCSPC	Neck top-down 1	512	SlimNeck fusion block
VoV-GSCSPC	Neck top-down 2	256	
GSConv	Bottom-up downsample 1	256	k=3, s=2
GSConv	Bottom-up downsample 2	512	
EMA	After P3/8	256	before Detect
EMA	After P4/16	512	
EMA	After P5/32	1024	

### 2.2. C3k2_DG module

**Fig 2:**
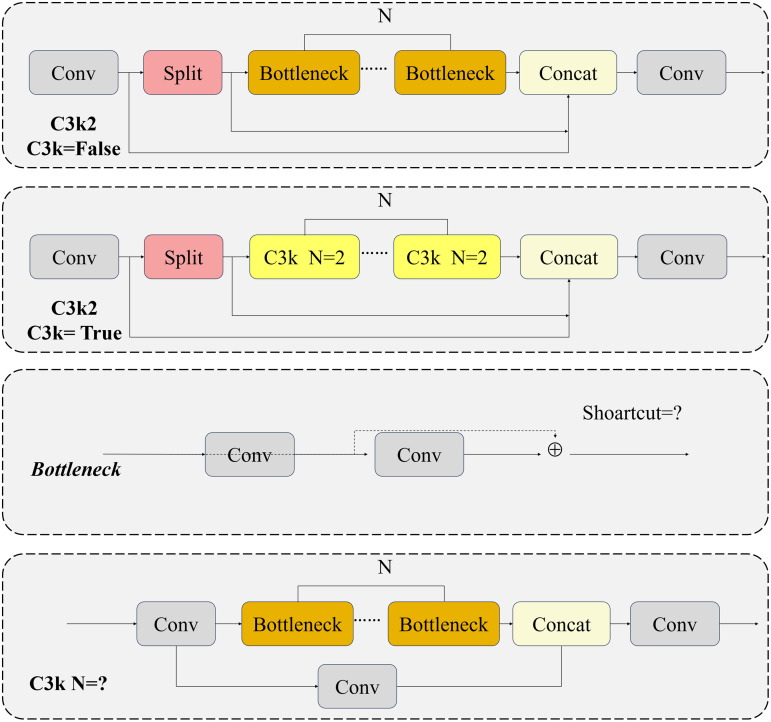
The structure of C3k2.

The conventional C3k2 module relies on fixed-parameter convolution kernels, making it challenging to adapt to the dynamic illumination variations in on-road scenarios and the complex properties of local features, such as occlusion interference under intricate road conditions and low-contrast speed bumps. This inherent limitation constrains the full exertion of its feature representation capability. The specific structure of the C3k2 module is shown in Fig 2.

**Fig 3:**
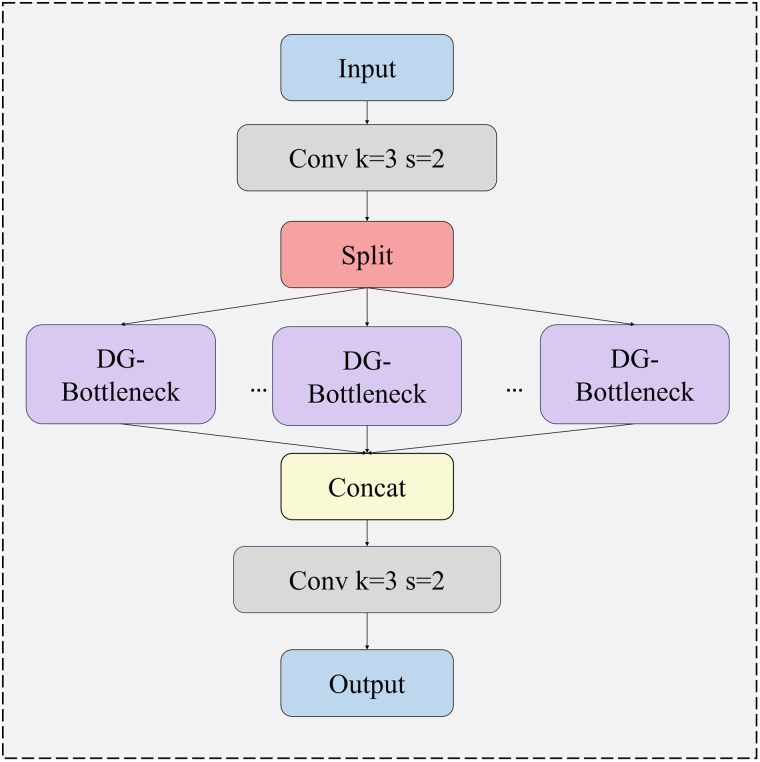
The structure of C3k2_DG.

When C3k2 = False, the Bottleneck module is employed to retain the conventional convolution operation. Conversely, when C3k2 = True, the C3k module is integrated to incorporate additional convolution layers, thereby enhancing the extraction of local features. To address the inadequacies in the model’s feature extraction capability and its poor adaptability to complex on-road scenarios during the detection of critical traffic elements, this study incorporates the DynamicConv dynamic convolution mechanism to optimize the C3k2 module [27]. By extending the DynamicConv mechanism and leveraging the architectural innovation of the GhostBottleneck module, the original Bottleneck module within the C3k2 structure is replaced. The newly designed Bottleneck module is named DG-Bottleneck, and the optimized C3k2_DG module is illustrated in Fig 3. Specifically, the DG-Bottleneck replaces fixed convolution with DynamicConv while retaining the residual/shortcut path.

The main difference between the proposed DG-Bottleneck and the original bottleneck lies in the convolutional transformation used for local feature extraction. In the original bottleneck, the feature transformation is performed by fixed convolution kernels whose weights are shared across all input samples after training. In contrast, DG-Bottleneck replaces the fixed convolutional transformation with a DynamicConv-based transformation.The C3k2_DG module adaptively selects or fuses distinct convolution kernels (referred to as experts) to process input data based on its inherent feature characteristics. While increasing the model parameter count, this module maintains a low floating-point operation (FLOP) count, striking a favorable balance between model capacity and computational efficiency. By embedding an attention mechanism, this dynamic learning strategy transforms the convolution operation from a simple linear mapping into a nonlinear process, thereby enhancing the representational capacity of convolution kernels and enabling more efficient extraction of discriminative image features [28]. The dynamic convolution calculation process is as follows.

Firstly, the weight coefficients of each expert convolution kernel are generated by the attention mechanism. The calculation formula is:


$$\begin{array}{c} \alpha =\text{soft}max(MLP(Pool(X))) \end{array}$$ (1)

In the formula, *X* denotes the input feature map, and α represents the attention coefficient of the convolution kernel. Specifically, a vector representation is obtained by performing global average pooling on *X.* Subsequently, a two-layer multi-layer perceptron (MLP) is activated via the Softmax function to output attention coefficients $\alpha_{\text{1}},\alpha_{\text{2}},\ldots ,\alpha_{n}$, which are used to weight each expert convolution kernel. In our implementation, Dynamic Convolution consists of K experts (with *K* = 4 in this study), labeled from expert 1 to expert *K*. Each expert uses a 3 × 3 convolution with stride 1 and padding 1 for spatial feature extraction, followed by batch normalization and SiLU activation,and this setting is fixed across layers. Then, α is employed to weight and fuse each expert convolution kernel, yielding the dynamic convolution kernel $Z^{\prime }$ [29].


$$\begin{array}{c} Z^{\prime }=\overset{K}{\underset{𝕚=\text{1}}{\Sigma }}\alpha_{i}Z_{i} \end{array}$$ (2)

Ultimately, the input feature map *X* undergoes convolution with the dynamic convolution kernel $Z^{\prime }$, yielding the output feature map *Y*.


$$\begin{array}{c} Y=X*Z^{\prime } \end{array}$$ (3)

**Fig 4:**
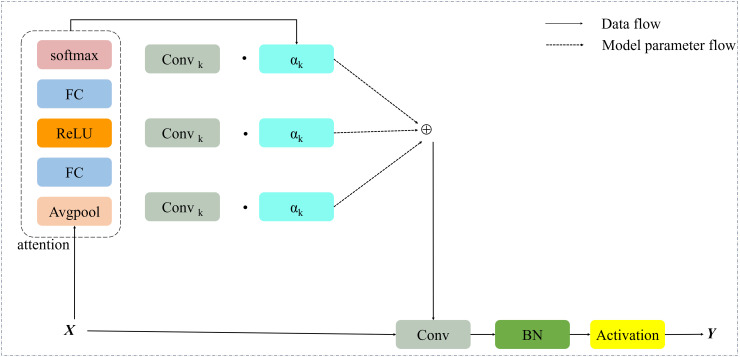
The structure of DynamicConv.

DynamicConv consists of k convolution kernels and adheres to the conventional architecture of convolutional neural networks, incorporating batch normalization BN and the rectified linear unit (ReLU) as its activation function. Its specific structural configuration is illustrated in Fig 4.

### 2.3. SlimNeck module

While lightweight models constructed using depthwise separable convolution (DSC) can effectively accelerate inference speed, their detection accuracy tends to degrade due to the disconnection of channel-wise information in input images during computation. To mitigate DSC’s inherent flaw, a series of schemes, such as MobileNets, ShuffleNets, and GhostNet, have been proposed. However, their channel-wise information interaction still relies on excessive computational resources, failing to achieve a comprehensive balance between lightweight properties and detection accuracy [30]. To address this gap, this study introduces a lightweight neck structure, SlimNeck, to build a real-time detection model with lower complexity and higher inference efficiency, well-suited for mobile computing platforms in scenarios such as autonomous driving.

Specifically, SlimNeck integrates the GSConv method by fusing and reorganizing features from standard convolution and depthwise separable convolution (DSC), thereby significantly reducing computational overhead while retaining feature representation comparable to that of standard convolution. Furthermore, an efficient cross-stage partial network module, VoV-GSCSP, based on GSConv, is designed. A one-time aggregation strategy is adopted to replace the C3k2 module in the YOLO11 neck, thereby enabling efficient multi-scale feature fusion and lightweight operation. This combined optimization enables SlimNeck to substantially reduce model parameters and computational overhead while preserving high detection accuracy, thereby providing a feasible, lightweight solution for real-time computer vision tasks on edge devices [31].

**Fig 5:**
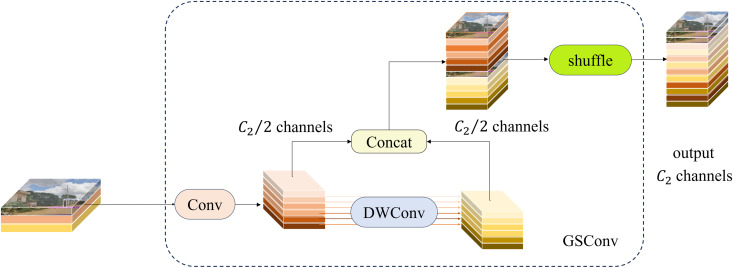
Structure of the GSConv Module. (a) GS Bottleneck Module, (b) VoV-GSCSP Bottleneck Module.

**Fig 6:**
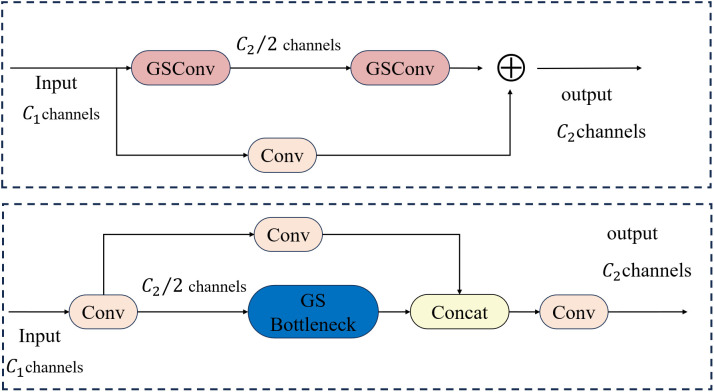
The Module of GS Bottleneck and VoV-GSCSP Bottleneck.

The architectural designs of GSConv and VoV-GSCSP are illustrated in Figs 5 and 6, respectively. As illustrated in Fig 5, the “GConv” module comprises three components. Specifically, “DWConv” denotes depthwise separable convolution, “Conv” represents standard convolution, “Concat” refers to the fusion of features from standard convolution and depthwise separable convolution, and “shuffle” denotes feature mixing via transposition, followed by the reconstruction of the corresponding channels.

For extracting key traffic elements, it is imperative to progressively project the spatial information encoded in the feature map onto the channel dimension, thereby enhancing the network’s capacity for semantic feature representation. However, each compression operation applied to the spatial dimension and expansion operation implemented on the channel dimension during this process inevitably results in the loss of partial semantic information. Specifically, channel-dense convolution (SC) preserves implicit inter-channel correlations, whereas channel-sparse convolution (DSC) severs cross-channel connections, thereby constraining its feature representation capability. To address this technical limitation, GSConv integrates output maps from standard convolution and depthwise separable convolution, enabling the implementation of SC-like operations with substantially reduced computational overhead while preserving inter-channel connections and minimizing semantic information loss.

### 2.4. Efficient multi-scale attention module

**Fig 7:**
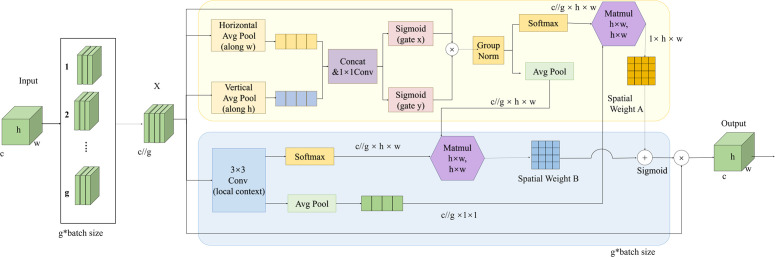
The structure of EMA.

In the backbone-neck integrated architecture of YOLO11, a hierarchical feature pyramid network (FPN) is used to fuse multi-scale features, thereby boosting the model’s ability to detect road targets across scales. However, cross-scale feature aggregation, despite enhancing feature richness, may inevitably introduce channel redundancy and semantic interference. Particularly for road-damage targets (e.g., Road Damages, Unsurfaced Road) characterized by irregular morphologies and intricate texture patterns, noise from irrelevant features tends to dilute the critical discriminative information essential for accurate detection. To optimize the multi-scale feature selection mechanism, this study integrates the Efficient Multi-scale Attention (EMA) module downstream of the three key feature levels (P3/8, P4/16, P5/32) within the neck network. The architecture of the EMA module is shown in Fig 7.

Given an input feature map $X\in R^{h\times w\times c}$ (where h, w, and c denote the height, width, and channel dimension of the feature map, respectively), the EMA module first partitions *X* into g sub-feature maps along the channel dimension to facilitate the learning of distinct semantic representations. This channel-wise grouping operation can be formally expressed as $X=[X_{\text{0}},X_{i},\ldots ,X_{g-\text{1}}]$, where $X_{i}\in R^{c//g\times h\times w}$. Subsequently, each group of sub-features is fed into two parallel branches to compute the inter-channel attention coefficients. Specifically, in the 1 × 1 convolution branch, bidirectional one-dimensional global average pooling is employed to separately encode spatial positional information along the horizontal and vertical directions, whereas the 3 × 3 convolution branch extracts local spatial fine-grained features via 3 × 3 convolution operations. Thereafter, two-dimensional global average pooling is applied to the outputs of the two parallel branches to encode global spatial information and reshape them into the corresponding dimensional forms, namely $\overset{\text{1}\times c//g}{\underset{\text{1}}{R}}$ and $\overset{c//g\times h\times w}{\underset{\text{2}}{R}}$. After linear transformation, the outputs of the two branches with matched dimensions are fused via the nonlinear Softmax function to generate a spatial attention map of size 1 × h × w. By performing element-wise operations on the outputs of parallel multi-scale processing branches, spatial information is aggregated across different scales [32]. Ultimately, the EMA module outputs a feature map with the same spatial dimensions as the input feature map *X*. This design allows the module to be directly integrated into the YOLO11 network architecture, which not only substantially enhances the model’s capability to represent features of multi-scale road targets but also avoids introducing additional model parameters.

## 3. Experimental environment and hyperparameters

### 3.1. Datasets and experimental environment

#### 3.1.1. Datasets.

The experimental dataset used in this study was obtained from the Kaggle platform and comprises 8,394 road scene images covering three core categories of “person-vehicle-road” elements. Specifically, the annotated targets include Pedestrian, light motor vehicles (LMV), heavy motor vehicles, Road Damages, Speed Bump, and Unsurfaced Road. These goals constitute a common potential obstacle source in the automatic driving scene. Accurate perception of them is a necessary prerequisite to ensure traffic efficiency, maintain road structure safety, and avoid accident risk.

**Fig 8:**
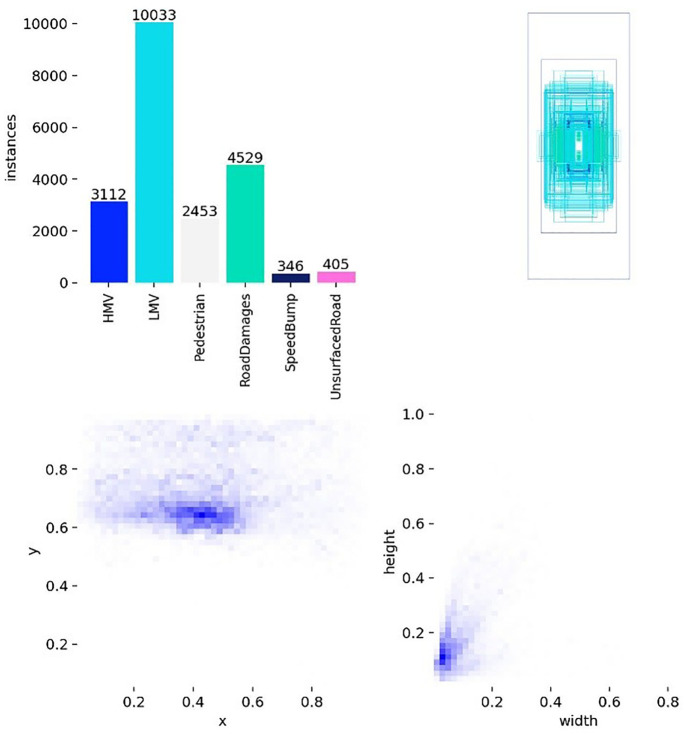
The characterization of the RAD dataset.

To better clarify the challenges posed by the RAD benchmark, we conducted statistical analysis on the annotation information from two dimensions: category distribution and target features. A statistical overview of the RAD dataset is provided in Fig 8.

The dataset contains six types of targets, with significant differences in the total number of instances, exhibiting a distinct long tail distribution feature. Among them, LMVs are the dominant category, with a total of 10033 instances. Road Damages and HMVs are second, with 4529 and 3112 instances, respectively. However, speed Bumps and Unsurfaced Roads belong to the tail sparse category, with only 346 and 405 instances, and the instance ratio between the front and rear categories is close to 29:1, which puts high demands on the model’s ability to handle class imbalance.

In terms of target scale, the width and height distribution of annotation boxes shows that small and medium-sized targets account for a higher proportion in the RAD dataset, especially in categories with weak visual saliency or limited training samples, such as speed bumps and pedestrians, where detection difficulty is more prominent. In addition, the spatial distribution of the target center point is mainly concentrated in the lower part of the image, which is consistent with the imaging pattern of the forward vehicle perspective. Although the dataset does not provide explicit labels at the scene level, such as weather and lighting, from a visual perspective, the diversity of road appearance, background complexity, and target combination methods is sufficient to construct a challenging evaluation environment.

For the training and evaluation of the follow-up model, t this study proportionally divided the dataset into a training set (5843 images), a validation set (1288 images), and a test set (1263 images). All input images are adjusted to a resolution of 640 × 640 pixels during the preprocessing stage. During the training phase, the batch size is set to 32, and the total number of training epochs is 300. The Stochastic Gradient Descent (SGD) optimizer is adopted, configured with an initial learning rate of 0.01, a momentum parameter of 0.937, and a weight decay coefficient of 0.0005. All images in the dataset are accompanied by corresponding annotation information, ensuring the validity of model training and evaluation.

#### 3.1.2. Experimental environment.

All experiments in this study were conducted on Windows 11, with PyTorch as the deep learning framework and PyCharm as the integrated development environment (IDE) for model training and experimental implementation.

During the experiments, all models were trained from scratch rather than initialized with pretrained weights. This setting was chosen to reduce the influence of external pretrained knowledge and to better isolate the contribution of the proposed architectural modifications under a unified training protocol. Specifically, by using the same from-scratch initialization, optimizer, input resolution, and training schedule for all models, we aimed to compare their intrinsic optimization and feature-learning ability on the RAD dataset itself. Nevertheless, pretrained initialization is a common practice in object detection and may benefit different detectors to different extents. Therefore, the from-scratch setting may not fully reflect the standard comparison setting used in the literature.

Moreover, all experiments were conducted with the same hyperparameter configuration for both the training and validation procedures. Detailed specifications of the experimental environment are presented in Table 2.

**Table 2:** Experimental environment configuration.

Experimental environment	Version
operating system	Windows11
GPU	NVIDIA GeForce RTX4060
GPU memory	8G
processor	Intel(R) Core (TM) i7-13650HX
programming language	Python 3.9.22
deep learning framework	Pytorch 2.0.0
CUDA	11.8

### 3.2. Evaluation metrics

For the key traffic element detection task, to comprehensively evaluate the detection accuracy, missing detection risk, and real-time inference performance of the model in complex road scenarios, the mean Average Precision (mAP), Precision (P), Recall (R), number of model parameters (Params), floating-point operations per second (FLOPs), and frames per second (FPS) are typically adopted as the core evaluation metrics. Specifically, the number of model parameters (Params) and giga floating-point operations (GFLOPs) serve as quantitative metrics for characterizing the model’s scale and computational complexity, respectively. Meanwhile, FPS reflects the model’s inference throughput under the present experimental hardware setting. It provides only an indirect indication of runtime efficiency and does not by itself establish deployment capability on embedded or edge devices. In parallel, P, R, and mAP evaluate the detection performance of key traffic elements from distinct dimensions.

Precision measures the proportion of results predicted to be abnormal that are correct. The calculation formula is:


$$\begin{array}{c} P=\frac{TP}{TP+FP} \end{array}$$ (4)

The Recall is used to measure the fraction of the real abnormal targets that are successfully detected. The calculation formula is:


$$\begin{array}{c} R=\frac{TP}{TP+FN} \end{array}$$ (5)

Where TP denotes the number of true positives, and FP denotes the number of false positives.

Given that the detection of key traffic elements frequently involves simultaneous identification of multi-scale targets, coupled with the pervasive challenges of target occlusion, weak texture features, and cluttered background interference, the Precision P or R metrics derived under a single confidence threshold are insufficient to comprehensively characterize the overall performance of the detection model. Accordingly, Average Precision (AP) is typically used to quantify the comprehensive detection performance of an individual category across a range of confidence thresholds, while mAP, derived by averaging AP across all categories, serves as a holistic performance metric for multi-category detection tasks. The mAP can be expressed as the mean of all categories of APs:


$$\begin{array}{c} mAP=\frac{\text{1}}{K}\sum \overset{K}{\underset{k=\text{1}}{\mathrm{\backslash nolimits}}}{AP}_{k} \end{array}$$ (6)

Where K is the total number of categories, AP_*k*_ is the average accuracy of the detection target of category k.

## 4. Experimental results and analysis

### 4.1. Model convergence analysis

**Fig 9:**
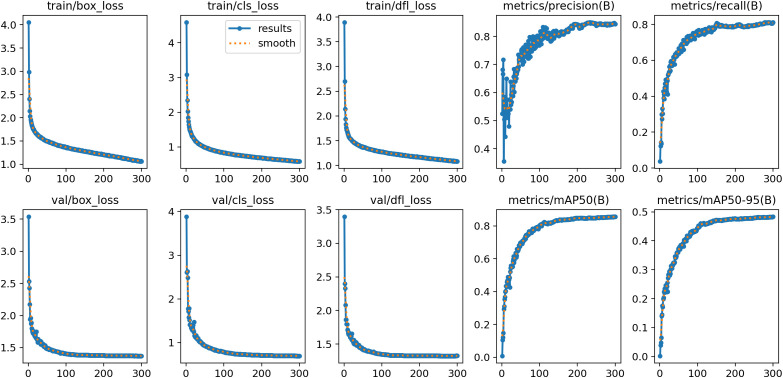
The convergence curve of DSE-YOLO11.

The convergence curves of the DSE-YOLO11 model on the Road Anomaly Detection (RAD) dataset are shown in Fig 9, where the curves labelled train and val show the trends of the training and validation sets during training. In the curves, box_loss denotes the bounding-box regression loss, which quantifies the positional deviation between predicted and ground-truth bounding boxes. refers to the classification loss, which reflects the model’s ability to accurately discriminate between target categories. Meanwhile, dlf_loss represents the distributional focal loss, which characterizes the effectiveness of regression distribution learning and further measures the discrepancies in shape and scale between the predicted and ground-truth bounding boxes. As observed during training, with increasing iterations, the DSE-YOLO11 model exhibited a rapid decline, followed by gradual stabilization, in box_loss, cls_loss, and dfl_loss on both the training and validation sets. Notably, no significant rebound in validation loss was observed throughout the training phase, indicating that the model did not suffer from substantial overfitting during optimization and that the overall training procedure was stable and reliable. Meanwhile, the Precision and Recall metrics exhibited a continuous upward trend and gradually entered a plateau phase in the late training stage. This observation indicates that the model’s detection capability improved as training progressed, ultimately converging to a sufficiently stable state. Furthermore, mAP50 and mAP50 ~ 95 quantify the model’s comprehensive detection accuracy at an intersection over union (IoU) threshold of 0.50 and across a multi-threshold interval from 0.50 to 0.95, respectively. The growth rates of the two metrics decelerate markedly in the late phase of training, indicating that the marginal gain in model performance diminishes as the number of training epochs increases. This phenomenon suggests that the model is approaching its performance ceiling on the target dataset. In summary, the DSE-YOLO11 model demonstrates excellent convergence and stable generalization on the Road Anomaly Detection (RAD) dataset. Its training process exhibits remarkable stability, with consistent improvements in key evaluation metrics, indicating that the proposed model possesses robust convergence characteristics, stable optimization behavior, and good experimental reliability on the RAD dataset.

### 4.2. Comparison of detection results between YOLO11n and DSE-YOLO11

The detection targets investigated in this study fall into three primary categories: vulnerable road users (pedestrians), road safety hazards (including Road Damage and Speed Bumps), and traffic operation-related elements. Given that the missed detection of the first two target categories would directly lead to elevated safety risks and maintenance costs, this study prioritizes recall rate in both the performance evaluation framework and the model architecture design. Meanwhile, to ensure that the detection outputs support subsequent positioning and quantitative analysis tasks, mAP50 ~ 95 is adopted as the positioning quality constraint metric, thereby guaranteeing that positioning performance does not degrade significantly while enhancing recall.

To comprehensively evaluate the category-level behavior of DSE-YOLO11, a comparison was conducted between the proposed model and the baseline YOLO11n, as summarized in Table 3. The results show that the effect of DSE-YOLO11 is not uniform across all categories, and the gains should therefore be interpreted in a class-dependent manner.

**Table 3:** Comparison of detection results between YOLO11n and DSE-YOLO11.

Model	Category	P	R	mAP50	mAP50 ~ 95
YOLO11n	LMV	0.884	0.884	0.957	0.628
	HMV	0.908	0.908	0.976	0.737
	Pedestrian	0.852	0.852	0.879	0.451
	Road Damages	0.657	0.657	0.612	0.249
	Speed Bumps	0.793	0.793	0.673	0.282
	Unsurfaced Road	0.769	0.769	0.767	0.404
DSE-YOLO11	LMV	**0.905**	**0.941**	**0.958**	0.628
	HMV	**0.926**	**0.954**	0.975	**0.738**
	Pedestrian	**0.882**	0.805	0.876	0.45
	Road Damages	**0.797**	0.514	**0.649**	0.273
	Speed Bumps	0.783	**0.904**	**0.891**	**0.429**
	Unsurfaced Road	**0.771**	0.749	**0.797**	0.379

Table 3 shows that the effect of DSE-YOLO11 is not uniform across all categories, and the gains should therefore be interpreted in a class-dependent manner. Notably, the most substantial improvement is observed in the detection of small-scale, low-contrast targets, particularly for the Speed Bumps category, where mAP50 improves by 21.8%. Concurrently, the mAP50 for the Road Damages increases by 14%. These two target categories are typically characterized by small object sizes and low contrast against the road background. The dynamic convolution module incorporated into the DSE-YOLO11 model effectively enhances the model’s capacity to capture fine-grained features and contextual information, thereby substantially boosting its detection accuracy and robustness on hard samples. For conventional large-scale targets (e.g., Light Motor Vehicles (LMVs) and Heavy Motor Vehicles (HMVs)), DSE-YOLO11 consistently maintains a robust competitive edge in terms of both precision and recall. Meanwhile, the mAP50 ~ 95 exhibits a modest but noticeable improvement. This empirical evidence corroborates that the proposed model enhancements do not compromise its baseline detection performance for general-purpose targets.

However, some categories exhibit clear trade-offs rather than consistent gains. In particular, Pedestrian recall decreases relative to YOLO11n, and Unsurfaced Road also shows a slight recall reduction. In addition, Road Damages presents a mixed pattern in which precision and mAP50 improve, whereas recall decreases. These results indicate that the proposed model does not uniformly improve all aspects of category-level detection performance. One possible explanation is that the enhanced feature-selection process may suppress some low-confidence true positives. Therefore, the proposed method should be interpreted as improving detection performance for selected difficult categories, especially Speed Bump, rather than providing uniform superiority across all classes. A more detailed class-wise analysis based on precision-recall curves and confusion patterns is provided in Section 4.4.3.

### 4.3. Ablation experimental analysis

To validate the efficacy of the improved method proposed in this study, YOLO11n is employed as the baseline model. Meanwhile, the performance contribution of each newly introduced module is systematically quantified in a step-by-step manner through rigorous ablation experiments. The corresponding experimental findings are summarized in Table 4. The “√” indicates that the corresponding module is added.

**Table 4:** Ablation experiment.

No.	Module			P	R	mAP50	mAP50 ~ 95	Params(M)	FLOPs(G)
	C3K2_DG	SlimNeck	EMA						
①				0.81	0.744	0.81	0.459	2.58	6.3
②	√			0.853	0.791	0.851	0.484	2.90	7.1
③		√		0.836	0.793	0.852	0.486	2.48	5.8
④			√	0.805	0.752	0.8	0.455	2.58	6.4
⑤	√	√		0.816	0.806	0.846	0.482	2.80	6.9
⑥	√	√	√	0.844	0.811	0.856	0.483	2.96	7.1

From the results of ablation experiments, it can be seen that:

(1) In Experiment ②, following the replacement of the C3k2 module with the C3k2_DG module in the backbone network, the precision, recall, and mAP50 metrics of the model exhibit respective increases of 4.3%, 4.7%, and 4.1%. This result demonstrates that the C3k2_DG module plays a pivotal role in boosting the model’s feature representation, while simultaneously enhancing the separability of targets with diverse morphological characteristics and varying scales in complex road scenarios, with only a slight increase in FLOPS. In Experiment ③, after introducing VoV-GSCSP and GSConv in the Neck part, the model improves the recall rate and mAP50 ~ 95 while reducing the number of model parameters and computational cost, highlighting the module’s feature-fusion efficiency and lightweight advantages. In Experiment ④, following the incorporation of the EMA attention mechanism into the P3, P4, and P5 feature layers, the recall rate registered a 0.8% improvement, which demonstrates the potential of this module to enhance the model’s capability of focusing on key features.
(2) Experiment 5 achieves a high recall rate under the premise of good control of parameter quantity and calculation amount, indicating that the C3K2 _ DG module and SlimNeck complement each other in enhancing feature diversity and propagation efficiency. In Experiment 6, the optimal trade-off between model performance and computational efficiency was attained following the synergistic integration of the C3k2_DG, SlimNeck, and EMA modules. Relative to the baseline model, the model’s Precision (P), Recall (R), and mAP50 metrics increased by 3.4%, 6.7%, and 4.6 percentage points, respectively. Whereas computational complexity (GFLOPs) increased by only 12.7%, the final model parameter count (2.96M) remained lightweight.

The ablation results suggest that the three modules play complementary rather than identical roles. C3k2_DG provides the main gain in adaptive local feature extraction, SlimNeck improves multi-scale fusion efficiency with limited additional cost, and EMA contributes mainly as a refinement module. This interpretation also helps explain why EMA alone yields only modest gains but performs better when combined with the backbone and neck modifications: its effect appears to depend on the quality of the upstream features. Therefore, the advantage of the full model is more appropriately interpreted as a complementary interaction among feature extraction, feature fusion, and attention-based refinement, rather than as a fully verified additive or mechanistic synergy.

### 4.4. Analysis of comparative experiments

#### 4.4.1. Comparative experiments of different models.

To further validate the efficacy of the proposed improved model, a comprehensive comparative analysis is conducted on the DSE-YOLO11 model against a suite of state-of-the-art object detection models. This suite encompasses representative mainstream models, including Faster R-CNN and SSD, as well as advanced YOLO variants. The quantitative experimental outcomes generated from this rigorous comparative study are systematically summarized in Table 5.

**Table 5:** Quantitative comparison with representative object detection methods on the RAD dataset.

Model	Params(M)	FLOPs(G)	FPS	P	R	mAP50	mAP50 ~ 95
Faster-RCNN	41.76	268.76	16.12	0.504	0.690	0.766	–
SSD	35.64	69.72	36.16	0.667	0.397	0.650	–
RT-DETR [33]	32.00	103.5	42.1	0.8	0.801	0.839	0.477
PP-YOLOE [34]	6.24	17.4	30.9	–	–	0.795	0.447
YOLOX [35]	8.94	26.8	171	0.801	0.794	0.820	0.468
YOLOv5n	2.50	7.1	167.3	0.806	0.803	0.843	0.479
YOLOv8n	3.01	8.1	193.2	0.844	0.778	0.851	0.486
YOLOv10n	2.6	6.5	178.8	0.829	0.769	0.835	0.475
YOLO11n	2.58	6.3	174.5	0.810	0.744	0.810	0.443
YOLO12n	2.51	5.8	110.7	0.842	0.762	0.823	0.472
DSE-YOLO11	2.96	7.1	95.2	0.844	**0.811**	**0.856**	0.483

As shown in Table 5, DSE-YOLO11 achieves the strongest overall balance among the compared models on RAD, with P = 0.844, R = 0.811, and mAP50 = 0.856. Compared with heavier detectors such as Faster R-CNN and RT-DETR, it reaches competitive or better detection performance with much lower model complexity. Compared with lightweight YOLO-family baselines, it provides the highest recall and mAP50, indicating that the proposed modifications improve target detectability on this benchmark.

Among the representative modern detectors, RT-DETR achieved relatively competitive results, with a recall of 0.801 and an mAP50 of 0.839. However, its parameter count and computational cost remained substantially higher than those of DSE-YOLO11, reaching 32.00 M parameters and 103.5 G FLOPs, respectively. These results indicate that although RT-DETR provides a good balance between detection accuracy and robustness, DSE-YOLO11 achieved higher recall and mAP50 with much lower model complexity, suggesting a more favorable trade-off for lightweight traffic-scene deployment.

PP-YOLOE also showed relatively low complexity, with only 6.24 M parameters and 17.4 G FLOPs, indicating good computational efficiency. However, its mAP50 and mAP50–95 were 0.795 and 0.447, respectively, both lower than those of DSE-YOLO11. In addition, precision and recall values were not reported for PP-YOLOE in this comparison, which limits a more complete assessment of its detection characteristics. Nevertheless, based on the available metrics, DSE-YOLO11 achieved stronger overall detection accuracy while remaining within a lightweight model range.

YOLOX demonstrated strong real-time capability, with an inference speed of 171 FPS, and achieved relatively balanced precision and recall values of 0.801 and 0.794, respectively. However, its mAP50 and mAP50–95 were 0.820 and 0.468, both below the corresponding values of DSE-YOLO11. Compared with YOLOX, DSE-YOLO11 improved recall, mAP50, and mAP50–95, although at a lower inference speed. This result suggests that the proposed model places greater emphasis on reducing missed detections and improving detection robustness in complex traffic scenes, rather than maximizing throughput alone.

Among the YOLO-series models, DSE-YOLO11 achieved the highest recall and mAP50 while maintaining high precision. Compared with YOLOv8n, DSE-YOLO11 improved recall by 0.033 and mAP50 by 0.005, with the same precision value of 0.844. Compared with the baseline YOLO11n, DSE-YOLO11 improved precision, recall, and mAP50 by 0.034, 0.067, and 0.046, respectively. These findings indicate that the proposed architectural modifications improved feature representation and enhanced detection performance for key traffic elements.

This gain comes with a clear trade-off. Relative to YOLO11n, DSE-YOLO11 increases Params and GFLOPs only moderately but reduces inference speed from 174.5 FPS to 95.2 FPS on the RTX4060 platform. The model is therefore better interpreted as favoring missed-detection reduction over maximum throughput. Because all efficiency measurements were obtained on a discrete GPU, they should not be taken as direct evidence of embedded or edge deployment capability.

It should also be noted that DSE-YOLO11 achieved an mAP50 ~ 95 of 0.483, which was slightly lower than the 0.486 obtained by YOLOv8n. This suggests that the proposed improvements mainly enhanced target detectability and robustness at an IoU threshold of 0.5, whereas improvements in fine-grained localization under stricter IoU thresholds were limited. Given that the difference in mAP50 ~ 95 between the two models was only 0.003, the results indicate that DSE-YOLO11 improved recall and overall detection quality without causing substantial degradation in localization performance.

**Fig 10:**
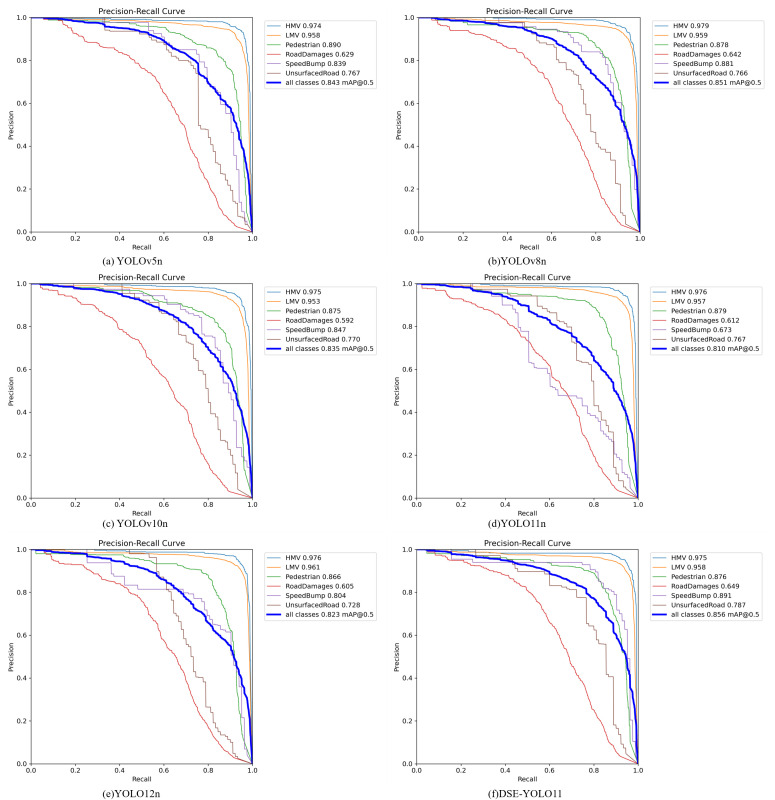
Precision-recall curves of representative detection models on the RAD dataset.

To further compare the threshold robustness and overall detection behavior of representative models, the precision-recall (P-R) curves on the RAD dataset are shown in Fig 10. Compared with the competing methods, DSE-YOLO11 maintains a more favorable precision-recall trade-off, especially in the medium-to-high recall range.

As shown in Fig 10, the proposed model achieves the highest overall detection accuracy, with an mAP50 of 0.856. Compared with YOLOv5, YOLOv8, YOLOv10, YOLO11, and YOLO12, the proposed model DSE-YOLO11 improves the all-class mAP50 by 1.3%, 0.5%, 2.1%, 4.6%, and 3.3%, respectively. This result indicates that the DSE-YOLO11 provides a more effective overall solution for multi-class object detection in complex traffic environments.

A category-level comparison further highlights the advantages of the proposed method on challenging targets. In particular, the AP values for Road Damages, Speed Bump, and Unsurfaced Road reach 0.649, 0.891, and 0.787, respectively, which are the highest among all competing models. These improvements suggest that the proposed model is more capable of capturing discriminative features for objects with irregular morphology, ambiguous boundaries, and severe background interference. By contrast, although several baseline models achieve comparable performance on relatively easy categories such as HMV and LMV, their performance declines more noticeably on difficult classes.

In addition, the P-R curves of the proposed method remain closer to the upper-right region of the plot over a wider recall range, indicating a better balance between precision and recall. This observation demonstrates that the DSE-YOLO11 not only improves the overall detection accuracy but also enhances robustness in complex scenarios. Therefore, the above results verify the effectiveness of the proposed approach and confirm its superiority over the compared YOLO series models.

#### 4.4.2. Comparative experiments adding different attention mechanisms.

To further validate the efficacy of the EMA attention mechanism module, a controlled comparative experiment is conducted in this study, where a suite of representative attention mechanism modules, including CBAM, CA, GAM, and ECA, are embedded in the identical network location, with their performance systematically contrasted against that of the EMA module. The quantitative outcomes derived from this group of controlled experiments are systematically tabulated in Table 6.

**Table 6:** Comparative results of different attention mechanisms.

Model	P	R	mAP50	Parameters	GFLOPS
YOLO11-D-S	0.816	0.806	0.846	2.80	6.9
YOLO11-D-S-CBAM	0.854	0.796	0.842	2.81	6.9
YOLO11-D-S-CA	0.823	0.808	0.843	2.80	6.9
YOLO11-D-S-GAM	0.861	0.779	0.846	2.91	8.2
YOLO11-D-S-EMA	0.844	**0.811**	**0.856**	2.96	7.1
YOLO11-D-S-ECA	0.830	0.801	0.848	2.81	6.9

Note: “D” stands for the DynamicConv module, “S” stands for the SlimNeck module.

According to the quantitative data in Table 5, integrating attention mechanisms has yielded varying degrees of improvement in the model’s overall performance relative to the baseline architecture. Among all the evaluated models, the one incorporating the EMA module achieves the best performance, attaining a peak mAP50 of 0.856 while maintaining high detection precision (0.844) and achieving the optimal recall rate (0.811). This finding demonstrates that the EMA module significantly enhances the model’s comprehensive detection performance for targets of varying scales in dynamic, complex road scenarios, thanks to its efficient multi-scale attention mechanism. In the key traffic element detection tasks studied, it is crucial to reduce the missed detection of speed bumps and road damage while maintaining the model’s efficiency. Although the number of parameters and computational cost are slightly higher than in the basic model, the increase is within an acceptable range, and a good balance between accuracy and efficiency is achieved. In contrast, other attention modules exhibit distinct trade-offs in target-detection tasks. For instance, the GAM module achieves the highest detection accuracy of 0.861, but this comes at the cost of a notable reduction in recall. Meanwhile, modules including CBAM, ECA, and CA excel in lightweight design optimization, but their comprehensive detection performance, as measured by the core metric mAP50, still falls short of that of the EMA-enhanced model. Therefore, EMA is selected as the best enhancement strategy.

#### 4.4.3. Class-wise PR-curve and confusion-pattern analysis.

**Fig 11:**
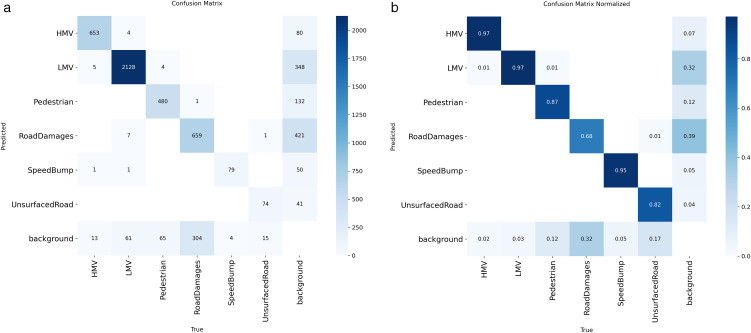
Confusion matrices of DSE-YOLO11 on the RAD dataset. (a) raw confusion matrix (b) confusion matrix normalized.

To provide a more balanced interpretation of the class-wise results, the class-wise precision-recall curves in Fig 10f and the confusion patterns in Fig 11a and b were further analyzed.

As shown in Fig 10f, the PR behavior differs substantially across categories. HMV and LMV achieve the most stable performance, with AP values of 0.975 and 0.958, respectively, indicating robust detection behavior. Speed Bump also shows a favorable PR profile, with an AP of 0.891, which is consistent with the marked gain observed in Table 2. These results suggest that the proposed architecture is particularly effective for this small and safety-critical target. By contrast, Road Damages remains the most challenging category. Its PR curve declines much more rapidly than those of the other categories, and its AP is the lowest (0.649), indicating that although the model improves mAP50 for this class, its detection robustness is still limited. Pedestrian and Unsurfaced Road show intermediate performance, but their PR curves are still noticeably weaker than those of the vehicle categories, especially in the higher-recall region. This observation is consistent with the class-wise trade-offs reported in Table 3.

**Fig 12:**
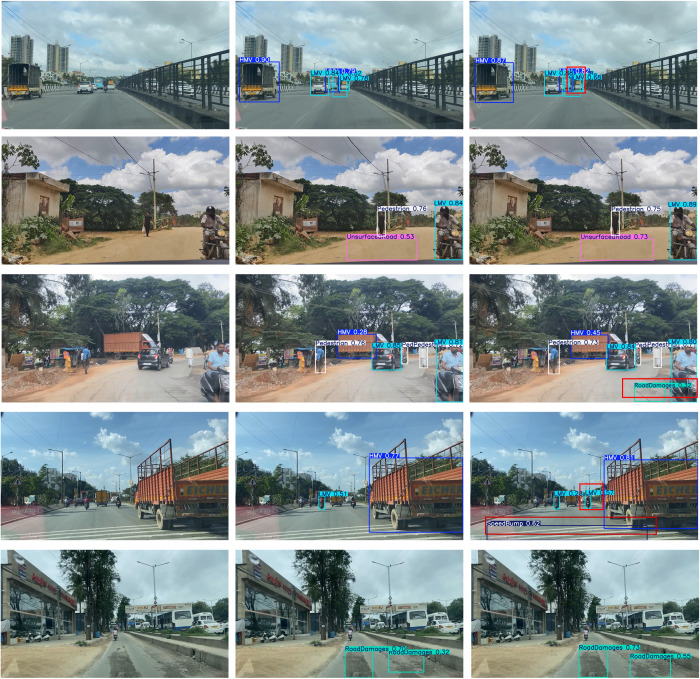
Detection effect comparison. (a) Original images, (c) DSE-YOLO11, (b) YOLO11n.

The confusion matrix analysis further clarifies the source of these trade-offs. As shown in Fig 12a and b, the dominant errors for the more difficult categories are primarily due to assignments to the background class, rather than strong confusion with other foreground categories. In the normalized confusion matrix, the proportion of ground-truth instances assigned to background is notably higher for Road Damages, Pedestrian, and Unsurfaced Road than for HMV, LMV, and Speed Bump. This indicates that the remaining limitations of the model are more strongly associated with insufficient sensitivity to weak, ambiguous, or irregular targets than with systematic inter-class confusion.

These results show that DSE-YOLO11 has clear advantages for some categories, particularly Speed Bump, but its gains are category-dependent and do not eliminate the detection difficulties of all traffic elements.

#### 4.4.4. External validation on the BDD100K dataset.

To provide evidence beyond a single benchmark, we additionally evaluated the RAD-pretrained models on BDD100K using overlapping categories only. The results are detailed in Table 7.

**Table 7:** External validation results on BDD100k.

Setting	Model	P	R	mAP50	mAP50 ~ 95
Zero-shot transfer	YOLO11n	0.0076	0.0718	0.0411	0.0253
Zero-shot transfer	DSE-YOLO11n	0.0743	0.0694	0.0483	0.0238
Transfer learning (50 epochs)	YOLO11n	0.429	0.272	0.279	0.155
Transfer learning (50 epochs)	DSE-YOLO11n	0.577	0.388	0.426	0.239

Note: Because the category definitions of RAD and BDD100K are not fully aligned, the comparison was conducted on overlapping categories only.

In the zero-shot setting, both YOLO11n and DSE-YOLO11 performed poorly, indicating a substantial domain gap between RAD and BDD100K. In the transfer-learning setting, however, DSE-YOLO11 consistently outperformed YOLO11n in Precision, Recall, mAP50, and mAP50-95. These results provide preliminary external support for the proposed architecture, but they should be interpreted cautiously because only one additional dataset and overlapping categories were considered.

### 4.5. Visualization of detection effect

To enable a more intuitive assessment of the detection performance of the proposed improved model, five representative sample images are carefully selected from the test dataset, encompassing a diverse range of typical road scenarios, namely, urban arterial roads, rural highways, and scenic thoroughfares, as illustrated in Fig 12. (a) Original images, (b) YOLO11n and (c) DSE-YOLO11

Group 1 presents detection results from complex urban road scenarios, where the detected targets typically exhibit medium-scale characteristics and often share color features with the surrounding roadside environment. Comparative analyses demonstrate that the proposed DSE-YOLO11 model substantially outperforms the baseline YOLO11 model in multi-category target detection tasks, specifically identifying more light motor vehicles (LMVs). Group 2 corresponds to unstructured rural road scenarios, where the detected targets span a scale range from small to medium. Specifically, for the detection task targeting Unsurfaced Road, the proposed DSE-YOLO11 model exhibits superior recognition performance, with detection confidence approximately 20 percentage points higher than that of the baseline YOLO11n model. The shared characteristic of the scenarios in Groups 3 and 4 is their high target density and partial-to-severe target occlusion, which place greater demands on the detection model’s robustness. In this context, comparative experiments demonstrate that the proposed DSE-YOLO11 model effectively mitigates the missed-detection issue inherent in the baseline YOLO11n model, accurately identifying and localizing Road Damage and Speed Bump targets, respectively. Group 5 corresponds to complex texture-dominated scenarios characterized by severe road-surface damage. The two models exhibit distinct performance disparities in such large-scale, texture-driven anomaly detection tasks. Specifically, compared with the baseline YOLO11n model, the proposed DSE-YOLO11 model achieves notably higher detection confidence for road surface damage targets. The detection confidence gains achieved for the three target categories, namely, light motor vehicles (LMVs), heavy motor vehicles (HMVs), and speed bumps, exhibit a consistent correlation with the recall rate enhancement trends derived from the head-to-head comparative experiments between the baseline YOLO11 and the proposed DSE-YOLO11 model, as systematically presented in Table 2.

## 5. Discussion

The present results indicate that DSE-YOLO11 is best understood as a task-specific lightweight redesign of YOLO11n rather than as a fundamentally new detector. Its main value lies in coordinating DynamicConv, SlimNeck, and EMA so that local feature extraction, cross-scale fusion, and feature refinement are improved in a way that suits the RAD task. Among the evaluated categories, the most notable improvement was observed for Speed Bump, indicating that the proposed design is particularly effective for targets that are small, ambiguous, or easily affected by background clutter.

However, the gains are category-dependent rather than uniform. Pedestrian and Unsurfaced Road remain more challenging, and Road Damages still exhibits a precision-recall trade-off. The PR-curve and confusion-matrix analyses suggest that many of the remaining errors arise from missed detections of weak or ambiguous targets rather than from strong inter-class confusion.

The external BDD100K analysis provides only preliminary support. Zero-shot transfer remains weak, indicating a clear domain gap, whereas transfer learning shows that the proposed architecture adapts better than YOLO11n under the tested setting. In addition, the efficiency results should be interpreted cautiously: the model remains lightweight in Params and GFLOPs, but the reported FPS was measured only on an RTX4060 GPU and does not establish deployment readiness on embedded hardware.

Several limitations remain. First, the current study does not include a dedicated sensitivity analysis for the number of dynamic experts, alternative EMA placements, or different degrees of SlimNeck replacement. Therefore, the present ablation results support a complementary interpretation of the three modules, but do not yet fully establish the mechanism of their interaction. Second, the comparative experiments were conducted under a from-scratch training protocol for all models. This design was adopted to provide a controlled comparison of architectural modifications on the RAD dataset, but it does not fully match the standard pretrained setting commonly used in object detection. Consequently, some baseline detectors may be affected more than others, and the reported absolute performance should be interpreted with caution. The current comparisons are therefore better understood as controlled from-scratch architecture comparisons rather than direct substitutes for standard pretrained benchmarks. Third, the study does not report device-side deployment metrics such as latency, runtime memory usage, or energy consumption. Therefore, the current evidence supports effectiveness on RAD and preliminary external adaptability, but not broad deployment or universal generalization claims.

## 6. Conclusion

In this study, we proposed DSE-YOLO11, a RAD-oriented lightweight redesign of YOLO11n for key traffic-element detection in complex road scenes. Rather than introducing new primitive modules, the method improves detection robustness by coordinating DynamicConv, SlimNeck, and EMA in a stage-wise manner. On the RAD benchmark, DSE-YOLO11 improves recall and mAP50 while keeping model complexity relatively low. Additional experiments on BDD100K provide preliminary external support, although broader validation is still needed.

Overall, the current results support DSE-YOLO11 as an effective architecture for the RAD task and as a promising basis for further study. Future work will focus on broader multi-dataset validation and deployment-oriented optimization, and report hardware-dependent metrics such as end-to-end latency, peak memory usage, and energy consumption. Additionally, it should include both pretrained-setting comparisons and hardware-specific evaluation.

## References

[1] Li G, Ji S, Cheng W, Wang W, Yan X, Yang Z, et al. A new framework for modelling traffic conflict interval time based on correlated random parameter duration model with heterogeneity in means and variances. Accid Anal Prev. 2026;224:108282. doi: 10.1016/j.aap.2025.108282 41166799

[2] Yang Y, Huang H, Li G, Han B, Yuan Z, Ma H. A systematic review of resilience assessment and enhancement of urban integrated transportation networks. J Transp Geogr. 2025;2025:104420. doi: 10.1016/j.jtrangeo.2025.104420

[3] Li G, Huang H, Han B, Jiang C, Pan Y, Zhou J. Exploring factors of E-bike rider’s hit-and-run behavior of E-bike crashes on urban and rural roads using Light Gradient Boosting machine and SHapley additive exPlanations. Transport Lett. 2026:1–19. doi: 10.1080/19427867.2026.2631120

[4] Hao Y, Li G, Lu J, Cheng W, Yuan Q, Yao Z. Knowledge graph-based safety risk evaluation method for hazardous behaviors of road transport vehicles. Traffic Inj Prev. 2025;:1–9. doi: 10.1080/15389588.2025.2540554 40833193

[5] Preethi P, Suresh RP, Sathwik HJ, Suman S, Mutasim M, Yashwin S. Development and evaluation of a comprehensive dataset for pothole depth estimation of Indian roads using smartphone camera approach. Singapore: Springer Nature Singapore; 2024.

[6] Girshick B, Donahue J, Darrell T, Malik J. Rich Feature Hierarchies for Accurate Object Detection and Semantic Segmentation. In: 2014 IEEE Conference on Computer Vision and Pattern Recognition. 2013. pp. 580–7.

[7] Ren S, He K, Girshick R, Sun J. Faster R-CNN: Towards Real-Time Object Detection with Region Proposal Networks. IEEE Trans Pattern Anal Mach Intell. 2017;39(6):1137–49. doi: 10.1109/TPAMI.2016.2577031 27295650

[8] Liu W, Anguelov D, Erhan D, Szegedy C, Reed S, Fu C-Y, et al. SSD: Single Shot MultiBox Detector. Cham: Springer International Publishing; 2016.

[9] Redmon J, Divvala SK, Girshick RB, Farhadi A. You Only Look Once: Unified, Real-Time Object Detection. CoRR. 2015.

[10] Xing TR, Zhang ZW, Guo Z, Chen SY. Radar deception jamming recognition based on multi-dimensional attention dynamic convolutional network. Intell Security. 2025;2025:71–81.

[11] Huo X, Sun G, Tian S, Wang Y, Yu L, Long J, et al. HiFuse: Hierarchical multi-scale feature fusion network for medical image classification. Biomed Signal Process Control. 2024;87:105534. doi: 10.1016/j.bspc.2023.105534

[12] Huang F, Zheng J, Liu X, Shen Y, Chen J. Polarization of road target detection under complex weather conditions. Sci Rep. 2024;14(1):30348. doi: 10.1038/s41598-024-80830-3 39639048 PMC11621122

[13] Qi G, Zhang Y, Wang K, Mazur N, Liu Y, Malaviya D. Small object detection method based on adaptive spatial parallel convolution and fast multi-scale fusion. Remote Sensing. 2022;14(2):420. doi: 10.3390/rs14020420

[14] Hong J, Ye K, Qiu S. Study on lightweight strategies for L-YOLO algorithm in road object detection. Sci Rep. 2025;15(1):7649. doi: 10.1038/s41598-025-92148-9 40038404 PMC11880549

[15] Mao G, Liang H, Yao Y, Wang L, Zhang N. ESPPNet: an efficient progressive spatial pyramid pooling network for real-time traffic object detection. IEEE Trans Automat Sci Eng. 2025;22:14048–61. doi: 10.1109/tase.2025.3558929

[16] Yuechen L, Yusheng C, Shixin J, Xiaoli W. A novel lightweight real-time traffic sign detection method based on an embedded device and YOLOv8. J Real-Time Image Process. 2024;24. doi: 10.1007/s11554-023-01403-7

[17] Gao Y, Qu Z, Wang S, Xia F. A lightweight neural network model of feature pyramid and attention mechanism for traffic object detection. IEEE Transac Intell Veh. 2024;:3422–35. doi: 10.1109/TIV.2023.3345271

[18] Yuan Z, Liu Z, Zhu C, Qi J, Zhao D. Object detection in remote sensing images via multi-feature pyramid network with receptive field block. Remote Sens. 2021;13(5):862. doi: 10.3390/rs13050862

[19] Xu H, Zheng WL, Liu FX, Li P, Wang RC. Unmanned aerial vehicle perspective small target recognition algorithm based on improved YOLOv5. Remote Sens. 2023. doi: 10.3390/rs15143583

[20] Zhang H, Liang M, Wang Y. YOLO-BS: a traffic sign detection algorithm based on YOLOv8. Sci Rep. 2025;15(1):7558. doi: 10.1038/s41598-025-88184-0 40038318 PMC11880478

[21] Yang L, Gu Y, Feng H. Multi-scale feature fusion and feature calibration with edge information enhancement for remote sensing object detection. Sci Rep. 2025;15(1):15371. doi: 10.1038/s41598-025-99835-7 40316719 PMC12048622

[22] Liu C, Zhang S, Hu M, Song Q. Object detection in remote sensing images based on adaptive multi-scale feature fusion method. Remote Sens. 2024;907.

[23] Shang R, Zhang J, Jiao L, Li Y, Marturi N, Stolkin R. Multi-scale adaptive feature fusion network for semantic segmentation in remote sensing images. Remote Sens. 2020;872.

[24] Wu Y, Mu X, Shi H, Hou M. An object detection model AAPW-YOLO for UAV remote sensing images based on adaptive convolution and reconstructed feature fusion. Sci Rep. 2025;15(1):16214. doi: 10.1038/s41598-025-00239-4 40346071 PMC12064822

[25] Xie J, Yuan B, Guo C, Li H, Wang F, Chu P, et al. KL-YOLO: a lightweight adaptive global feature enhancement network for small-object detection in low-altitude remote sensing imagery. IEEE Trans Instrum Meas. 2025;74:1–13. doi: 10.1109/tim.2025.357695742146727

[26] Kang M, Ting C-M, Ting FF, Phan RC-W. ASF-YOLO: A novel YOLO model with attentional scale sequence fusion for cell instance segmentation. Image Vis Comput. 2024;147:105057. doi: 10.1016/j.imavis.2024.105057

[27] Han K, Wang Y, Guo J, Wu E. ParameterNet: Parameters are all you need for large-scale visual pretraining of mobile networks. 2024.

[28] Tian C, Liu Z, Chen H, Dong F, Liu X, Lin C. A lightweight model for shine muscat grape detection in complex environments based on the YOLOv8 architecture. Agronomy. 2025;174.

[29] Fan Z, Wu Y, Liu W, Chen M, Qiu Z. LG-YOLOv8: A lightweight safety helmet detection algorithm combined with feature enhancement. Appl Sci. 2024;10141.

[30] Li H, Li J, Wei H, Liu Z, Zhan Z, Ren Q. Slim-neck by GSConv: a lightweight-design for real-time detector architectures. J Real-Time Image Process. 2024. doi: 10.1007/s11554-024-01436-6

[31] Chen KY, Du B, Wang YW, Wang GX, He JX. The real-time detection method for coal gangue based on YOLOv8s-GSC. J Real-Time Image Process. 2024. doi: 10.1007/s11554-024-01425-9

[32] Ouyang D, He S, Zhang G, Luo M, Guo H, Zhan J, et al. Efficient Multi-Scale Attention Module with Cross-Spatial Learning. In: ICASSP 2023 - 2023 IEEE International Conference on Acoustics, Speech and Signal Processing (ICASSP). 2023. pp. 1–5. doi: 10.1109/icassp49357.2023.10096516

[33] Zhao Y, Lv W, Xu S, Wei J, Wang G, Dang Q, et al. DETRs beat YOLOs on real-time object detection. 2024.

[34] Xu S, Wang X, Lv W, Chang Q, Cui C, Deng K, et al. PP-YOLOE: An evolved version of YOLO. ArXiv. 2022. https://arxiv.org/abs/2201.00001

[35] Zheng G, Liu S, Wang F, Li Z, Sun J. YOLOX: Exceeding YOLO Series in 2021. ArXiv. 2021.

